# Children's Cots

**Published:** 1892-11-12

**Authors:** 


### PRACTICAL DEPARTMENTS.

### CHILDREN'S COTS—III.

We have already referred to the absolute necessity which exists for the sides of cots used for sick or injured children to work easily and smoothly, and Mr. Gainsford's invention, which he showed at the Healtheries, certainly fulfilled this condition. The medal awarded to it at that exhibition was well deserved, for it is a very light and pretty as well as a useful cot. Probably the brass rods, up and down which the iron side pieces are arranged to slide, has something to do with the satisfactory working, but, anyhow, the result is admirable. We believe that Dr. Steele, of Guy's, took great interest in the special construction of this cot, about which he and Mr. Gainsford consulted, and it is now in use at many London and provincial hospitals. Since Mr. Gainsford's retirement from business the cot can be procured from Messrs. Tarn, Borough, London. To make this or any other cot quite complete for institution use, the addition of an eyelet of brass or iron to encircle the upright rods which support the drapery of a "bronchitis tent" is desirable. This should be fixed on the iron rail which forms the corner of the bedstead, just below the knob, and a small socket to receive the lower end of the tent pole should be placed half-way down the aforesaid corner rail. By this simple and very inexpensive arrangement a set of light rods can be attached to any cot in a ward with ease and with speed, both of which are essential when dealing with such urgent cases of illness or accident, as require the rapid erection of a "steam tent."

The cots in the new wards at Great Ormond Street Children's Hospital have these eyelets, and are also provided with spring bottoms, whilst well-fitting boards are kept in readiness for placing under matresses when specially firm beds are needed for particular cases. The sides of these cots work up and down very easily. They are made at Warrington, and appear well suited in every respect for institutional use.

When children's bedsteads are of the convenient height prescribed by modern taste, it becomes advisable to have castors of moderate size, for if these are so large that the slightest touch sets the long-legged cot in motion, the child feels insecure. Every intelligent nurse and mother knows what harm any kind of fear will exercise on a sickly child, even when it is not suffering from nerve or bone disease; but in the latter case any alarms, however slight the predisposing causes, should be absolutely avoided.

We have hitherto restricted our remarks to cots which have sides, not only because they are safer for young children, but also on account of their convenience for invalids who can by their means be accommodated with a play or meal table. This consists in a board suspended firmly from the top bar on each side, and is made exactly to fit the width of the cot, sometimes being merely a strip of deal, which is laid straight across, having a narrow piece of wood at either end, which clips the side, and thus keeps itself steady. A better, though rather more expensive, shape is one which drops down, as shown in our last week's illustration of Mr. Stidolph's institution cot. This is particularly convenient for little patients who must not sit up in bed, as it brings playthings within easy reach of eyes and hands. Mr. Stidolph's bed-table has tiny india rubber rollers, which fit closely, but cannot possibly injure the smartest of painted or brass cots.

Many varieties of swinging cots are made for babies, and numbers of the patterns are excellent; the best are those which are fairly, but not too deep, with sufficiently wide bottoms to take the bedding comfortably. Of course, the framework should be of strong and simple construction, with no useless projections to harbour dust; and although these cots should swing easily, the area of the motion should be confined within moderate and safe limits.

